# Supplementary material for: Bacterial Communities in the Embryo of Maize Landraces: Relation with Susceptibility to Fusarium Ear Rot
Source: Microorganisms. 2021 Nov 19;9(11):2388. doi: 10.3390/microorganisms9112388 (PMC8621305; doi:10.3390/microorganisms9112388)
Supplement: Supplementary file 1 [file microorganisms-09-02388-s001.zip › Figure_S1_Passera_et_al.pptx]

## Slide 1
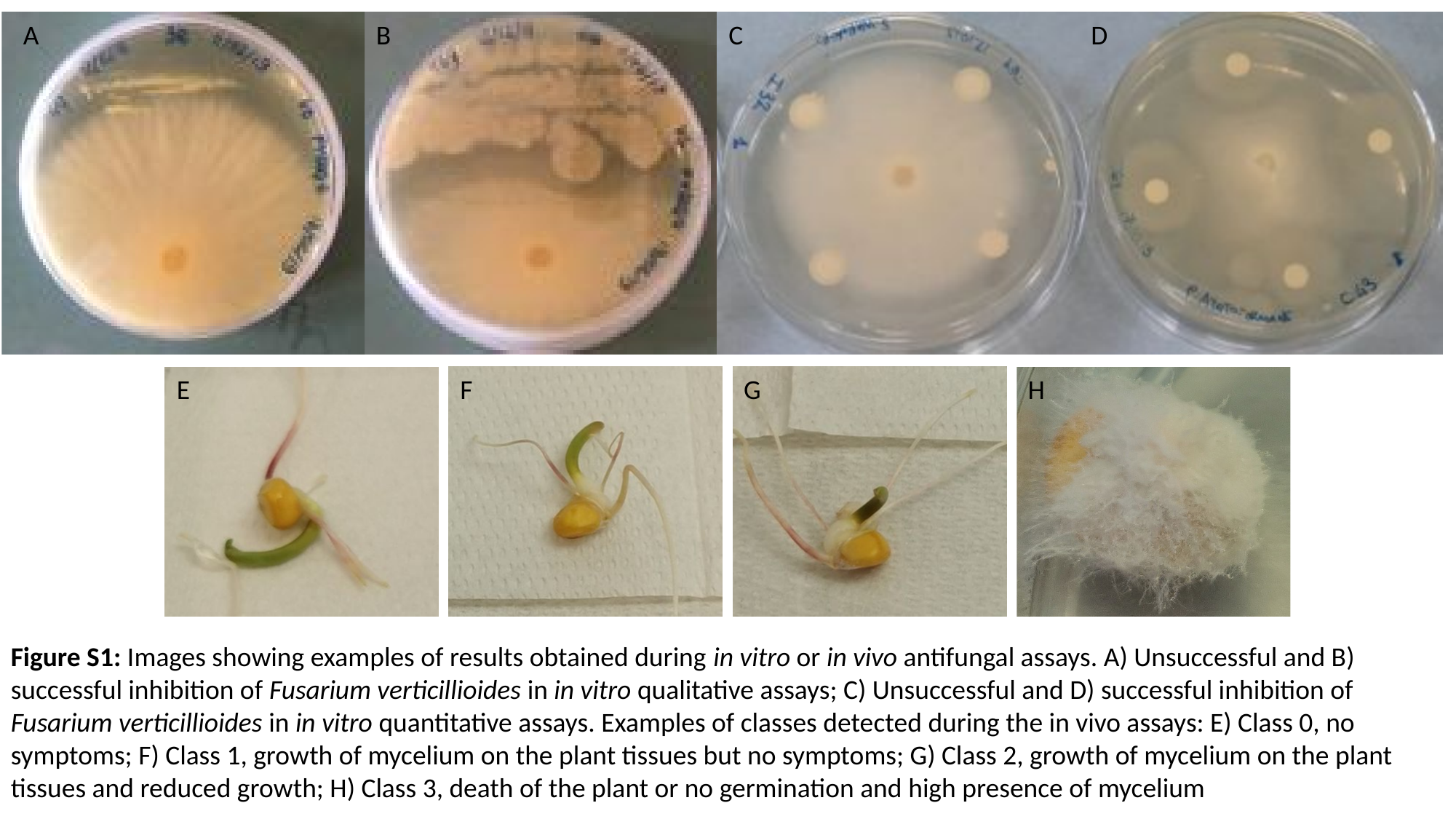

A
B
C
D
E
F
G
H
Figure S1: Images showing examples of results obtained during in vitro or in vivo antifungal assays. A) Unsuccessful and B) successful inhibition of Fusarium verticillioides in in vitro qualitative assays; C) Unsuccessful and D) successful inhibition of Fusarium verticillioides in in vitro quantitative assays. Examples of classes detected during the in vivo assays: E) Class 0, no symptoms; F) Class 1, growth of mycelium on the plant tissues but no symptoms; G) Class 2, growth of mycelium on the plant tissues and reduced growth; H) Class 3, death of the plant or no germination and high presence of mycelium
