# Supplementary material for: Bacterial Communities in the Embryo of Maize Landraces: Relation with Susceptibility to Fusarium Ear Rot
Source: Microorganisms. 2021 Nov 19;9(11):2388. doi: 10.3390/microorganisms9112388 (PMC8621305; doi:10.3390/microorganisms9112388)
Supplement: Supplementary file 1 [file microorganisms-09-02388-s001.zip › Figure_S2_Passera_et_al.pptx]

## Slide 1
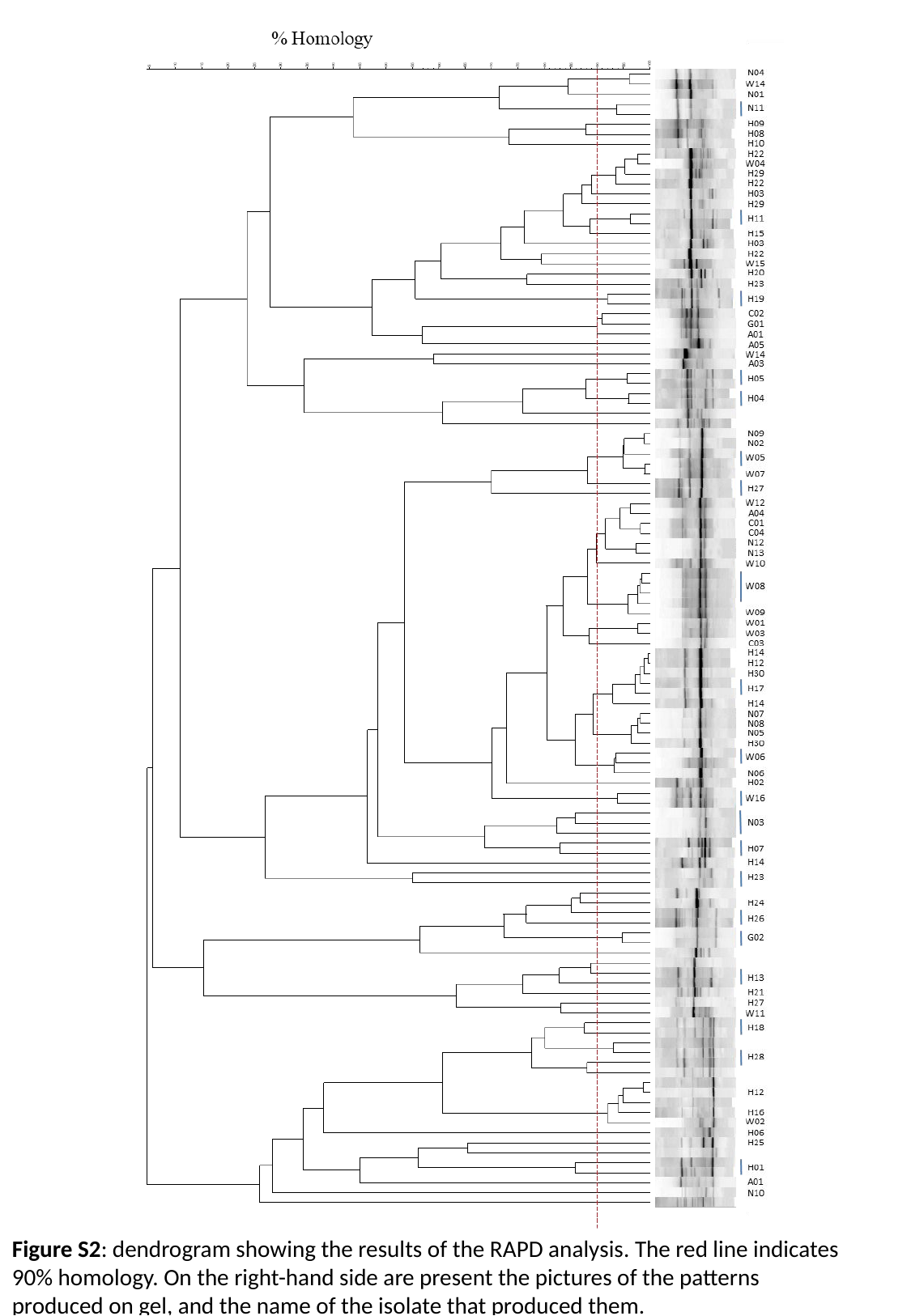

Figure S2: dendrogram showing the results of the RAPD analysis. The red line indicates 90% homology. On the right-hand side are present the pictures of the patterns produced on gel, and the name of the isolate that produced them.
